# Supplementary material for: Synthesis of Novel Acyl Derivatives of 3-(4,5,6,7-Tetrabromo-1H-benzimidazol-1-yl)propan-1-ols—Intracellular TBBi-Based CK2 Inhibitors with Proapoptotic Properties
Source: Int J Mol Sci. 2021 Jun 10;22(12):6261. doi: 10.3390/ijms22126261 (PMC8230474; doi:10.3390/ijms22126261)
Supplement: Supplementary file 1 [file ijms-22-06261-s001.zip › ijms-1240167-supplementary.pdf]

Supplementary material

Synthesis of Novel Acyl Derivatives of 3-(4,5,6,7-Tetrabromo-1*H*-benzimidazol-1-yl)propan-1-ols – Intracellular TBBi-based CK2 Inhibitors with Proapoptotic Properties

Konrad Chojnacki[1], Patrycja Wińska[1,*], Olena Karatsai[2], Mirosława Koronkiewicz[3], Małgorzata Milner-Krawczyk[1], Monika Wielechowska[1], Maria Jolanta Rędowicz,[2] Maria Bretner,[1] and Paweł Borowiecki[1]

[1] Chair of Drug and Cosmetics Biotechnology, Faculty of Chemistry, Warsaw University of Technology, 00-664 Warsaw, Poland
[2] Laboratory of Molecular Basis of Cell Motility, Nencki Institute of Experimental Biology, Polish Academy of Sciences, 02-093 Warsaw, Poland
[3] Department of Drug Biotechnology and Bioinformatics, National Medicines Institute, 00-725 Warsaw, Poland
* Correspondence: pwinska@ch.pw.edu.pl; Tel.: +48 222345573 (P.W.).

1. 1H NMR, 13C NMR and HRMS spectra:

1.1 3-(4,5,6,7-Tetrabromo-1H-benzimidazol-1-yl)propyl butanoate (3a)

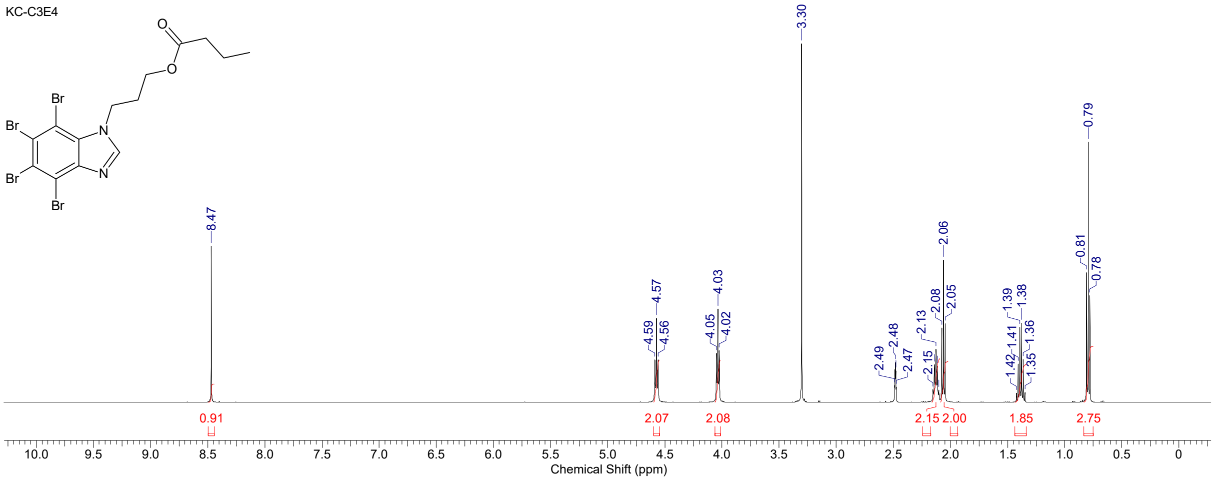

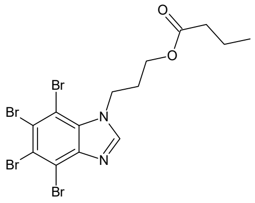

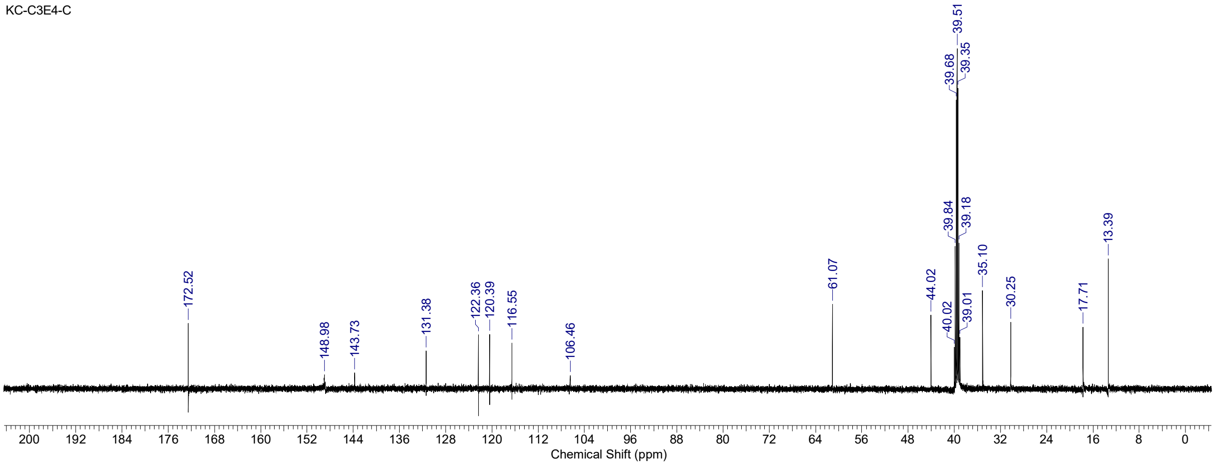

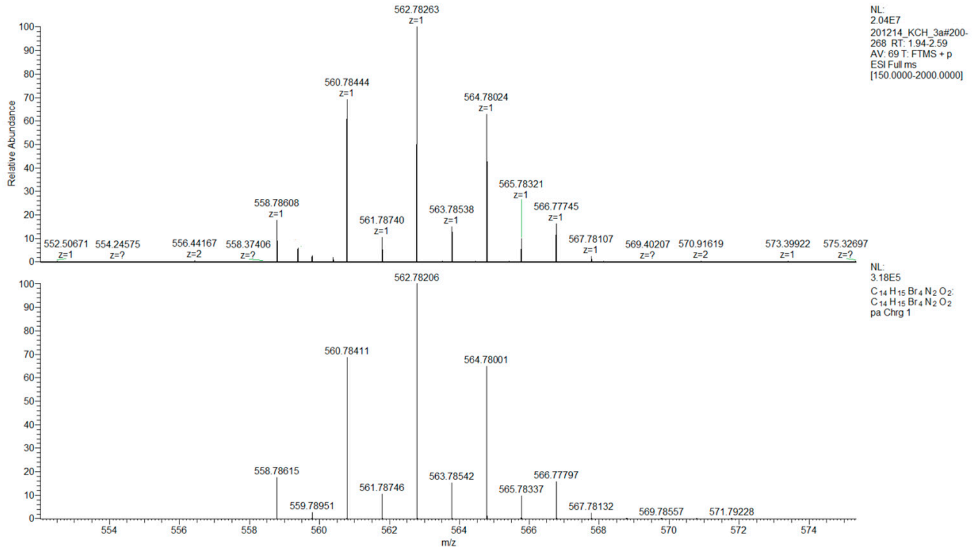

1.2 3-(4,5,6,7-Tetrabromo-1H-benzimidazol-1-yl)propyl hexanoate (3b)

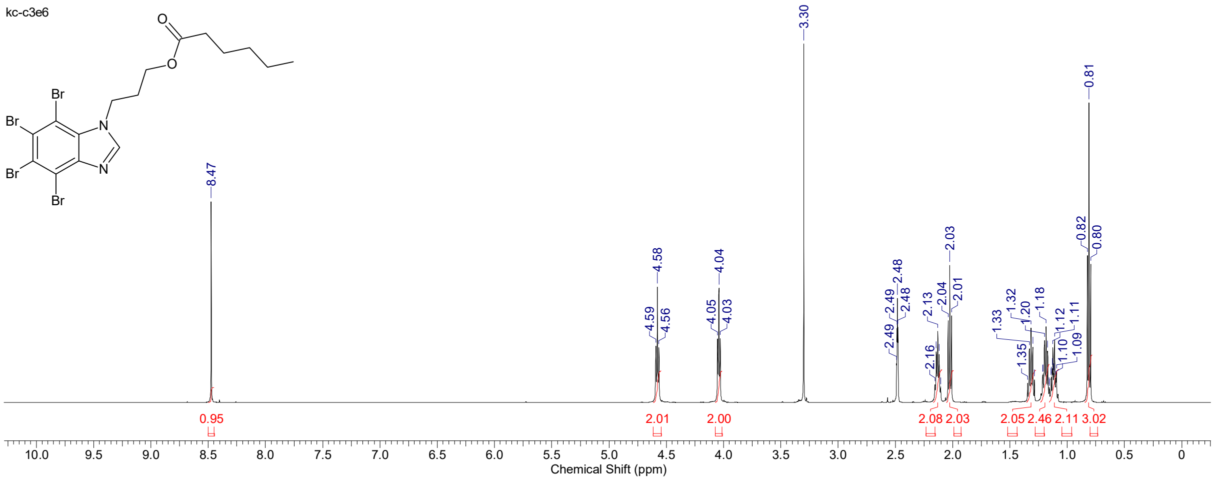

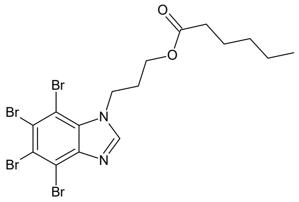

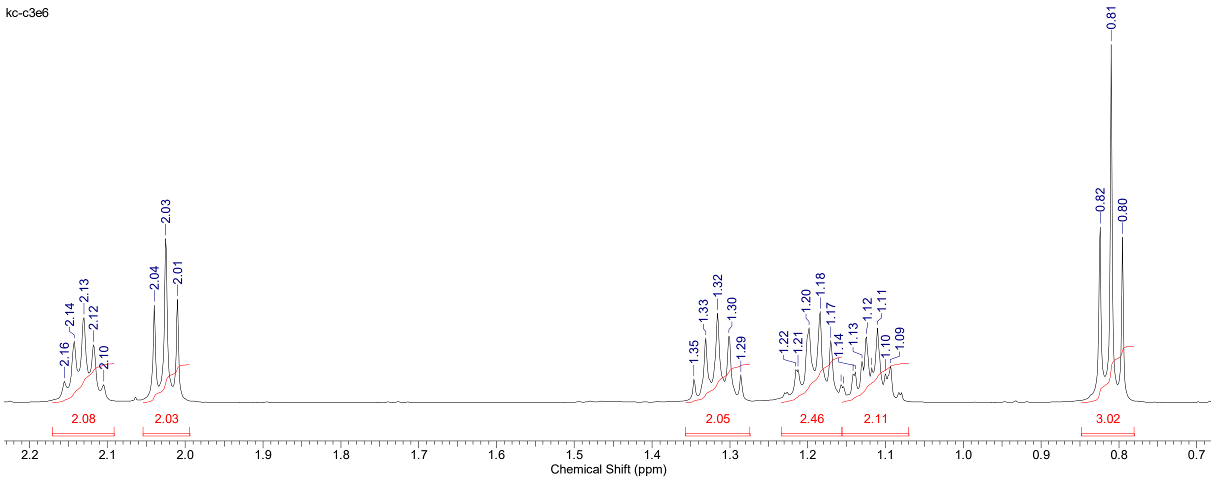

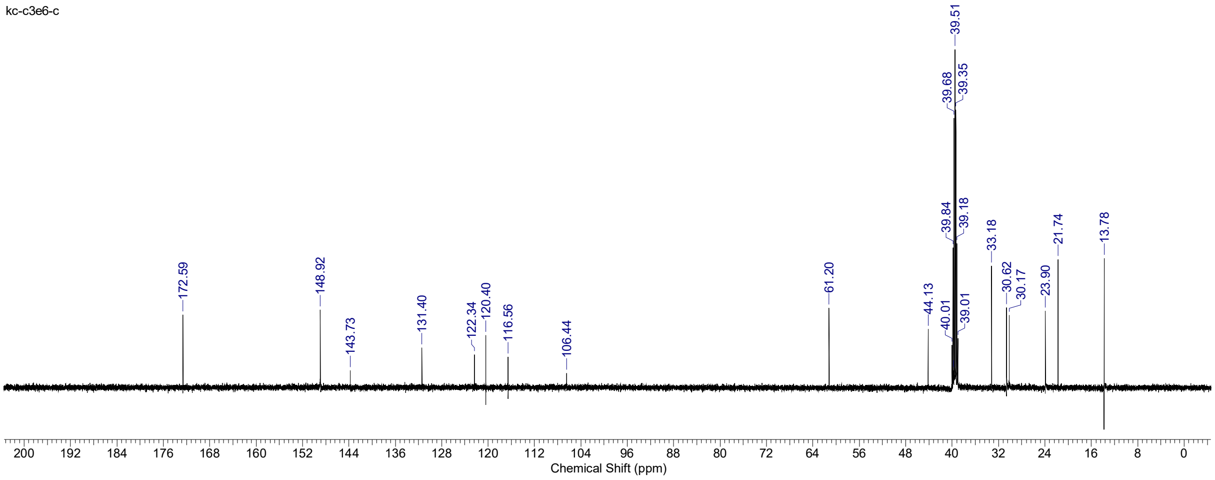

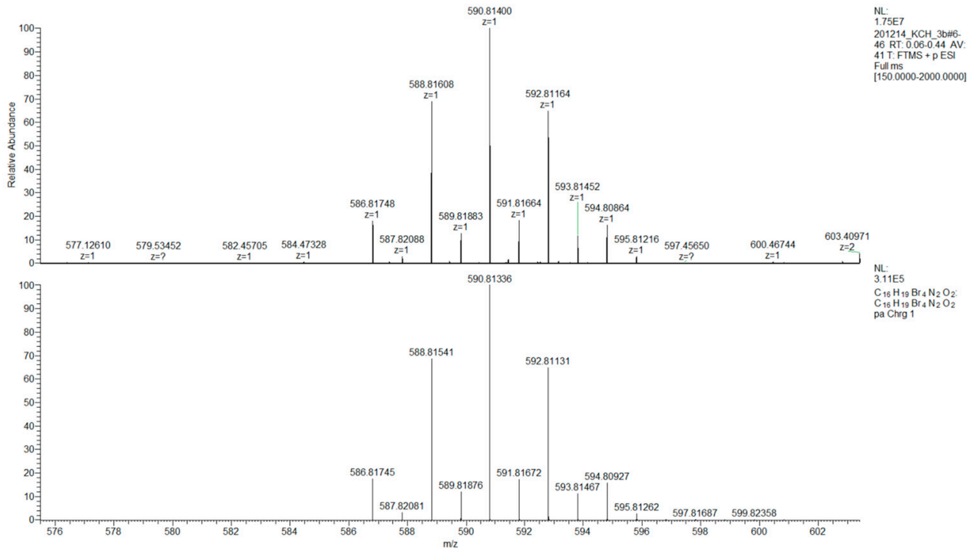

1.3 3-(4,5,6,7-Tetrabromo-1H-benzimidazol-1-yl)propyl octanoate (3c)

kc-c3e8

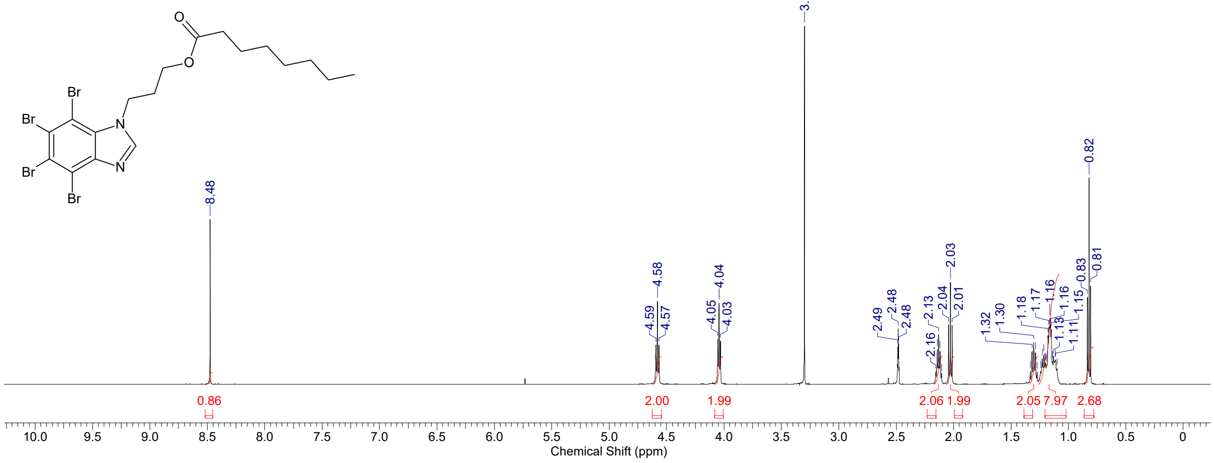

kc-c3e8

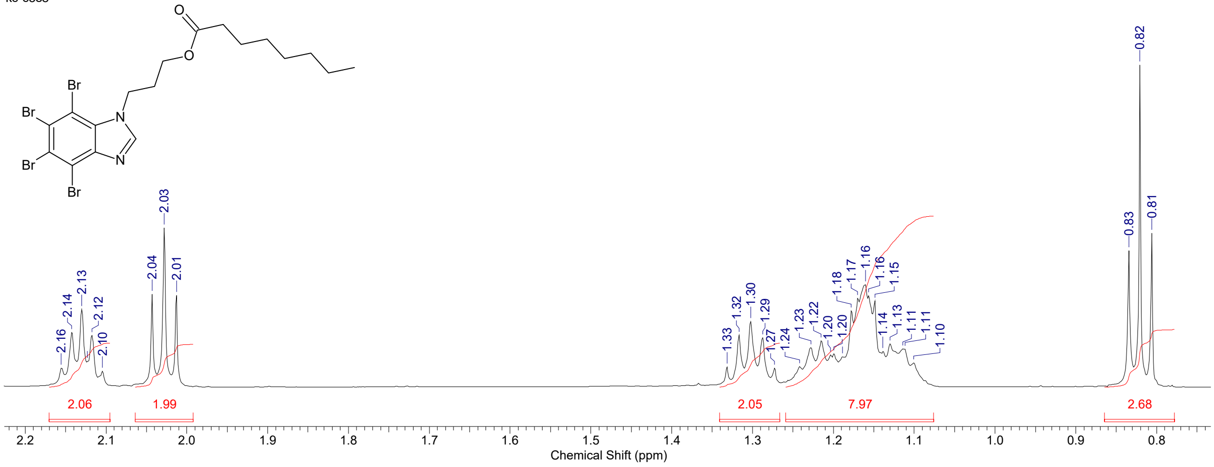

KC-C3E8-C

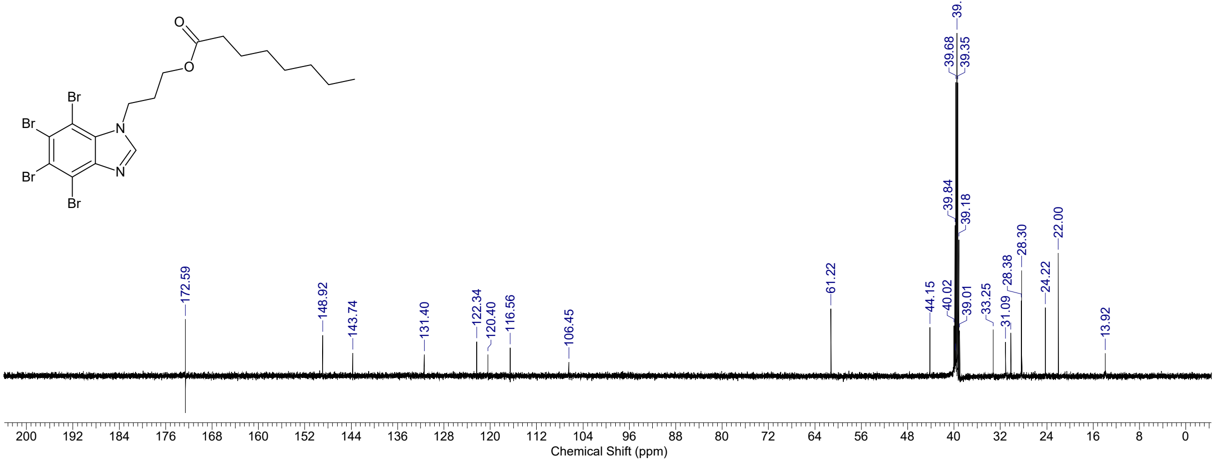

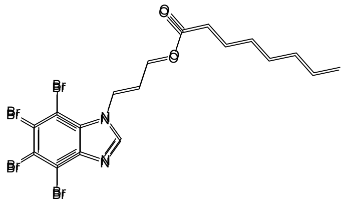

KC-C3E8-C

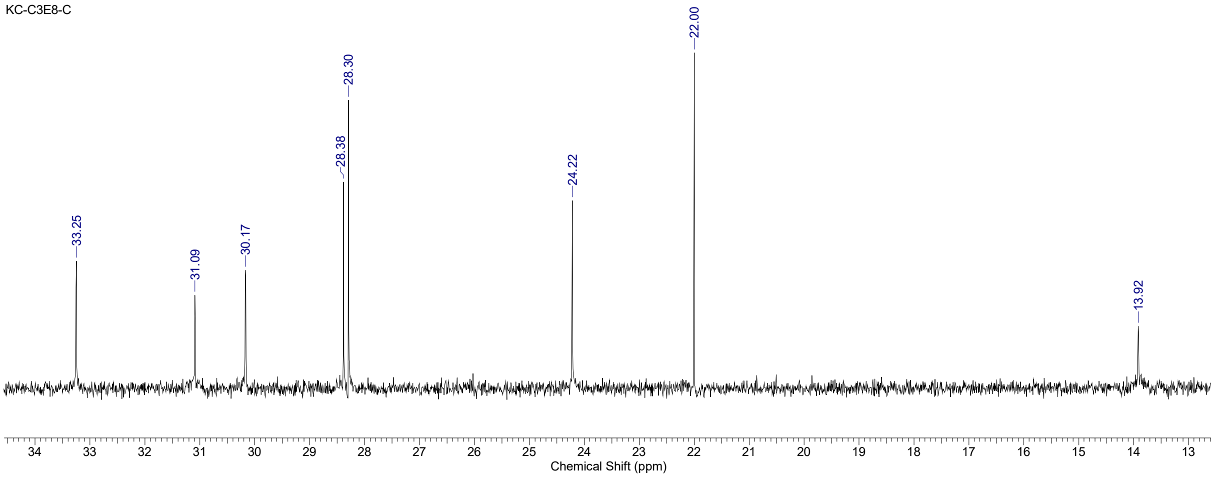

33.25
31.09
30.17
28.38
28.30
24.22
22.00
13.92
34 33 32 31 30 29 28 27 26 25 24 23 22 21 20 19 18 17 16 15 14 13
Chemical Shift (ppm)

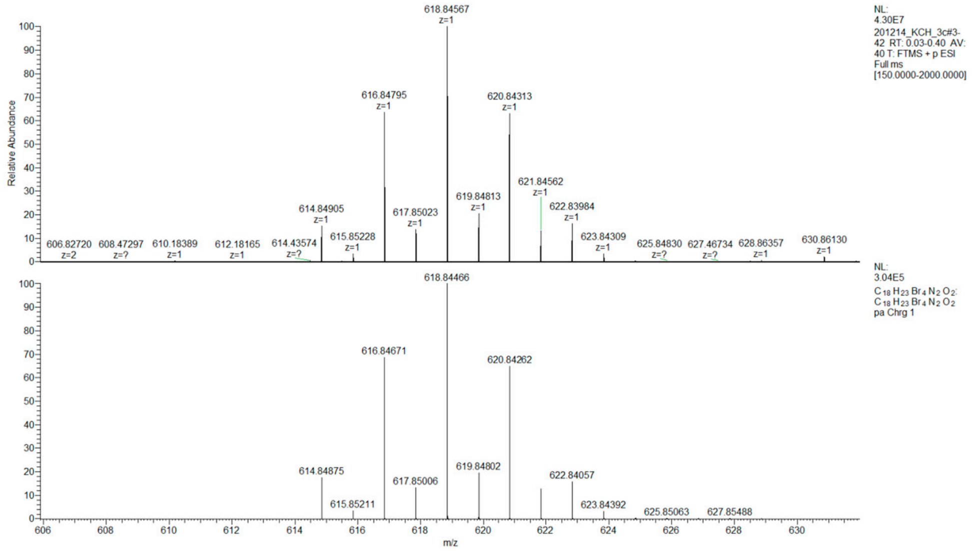

NL: 4.30E7
201214_KCH_3c#3-42 RT: 0.03-0.40 AV: 40 T: FTMS + p ESI Full ms [150.0000-2000.0000]
Relative Abundance
618.84567 z=1
616.84795 z=1
620.84313 z=1
621.84562 z=1
619.84813 z=1
614.84905 z=1
617.85023 z=1
622.83984 z=1
615.85228 z=1
623.84309 z=1
606.82720 z=2
608.47297 z=?
610.18389 z=1
612.18165 z=1
614.43574 z=?
625.84830 z=?
627.46734 z=?
628.86357 z=1
630.86130 z=1
NL: 3.04E5
C18 H23 Br4 N2 O2:
C18 H23 Br4 N2 O2
pa Chrg 1
618.84466
616.84671
620.84262
614.84875
619.84802
622.84057
617.85006
615.85211
623.84392
625.85063
627.85488
m/z

1.4 3-(4,5,6,7-Tetrabromo-1H-benzimidazol-1-yl)propyl dodecanoate (3d)

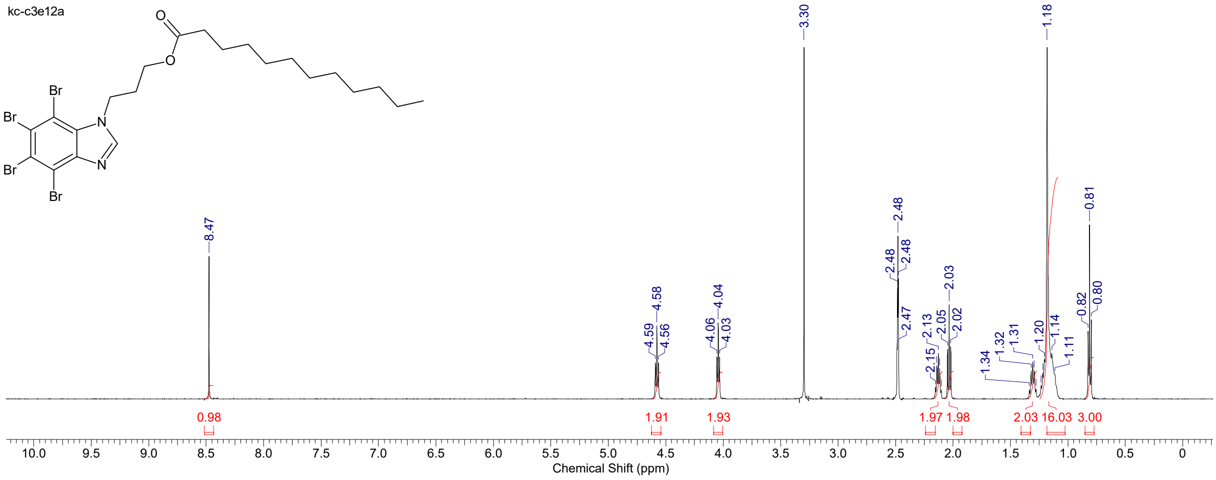

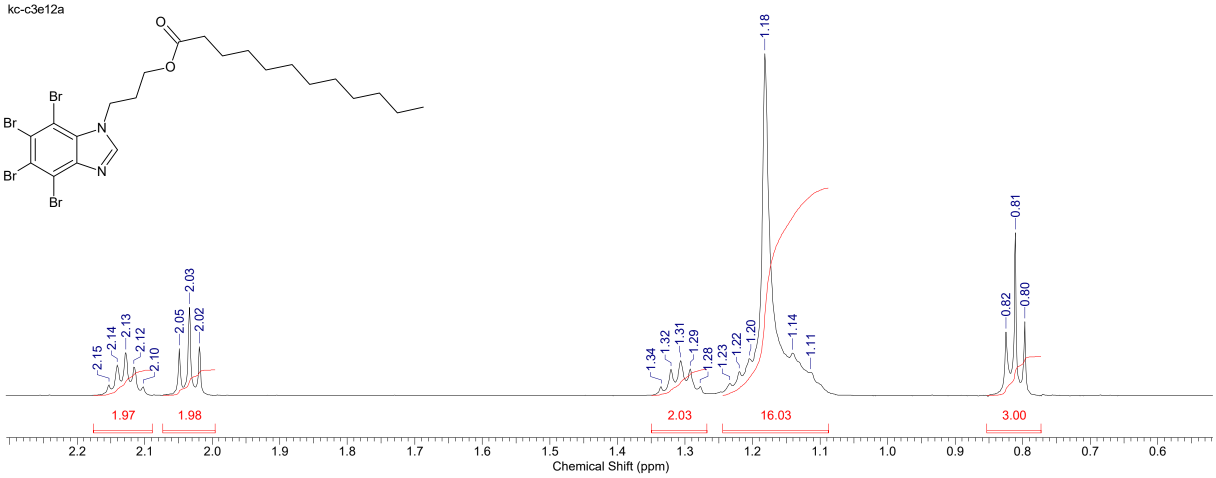

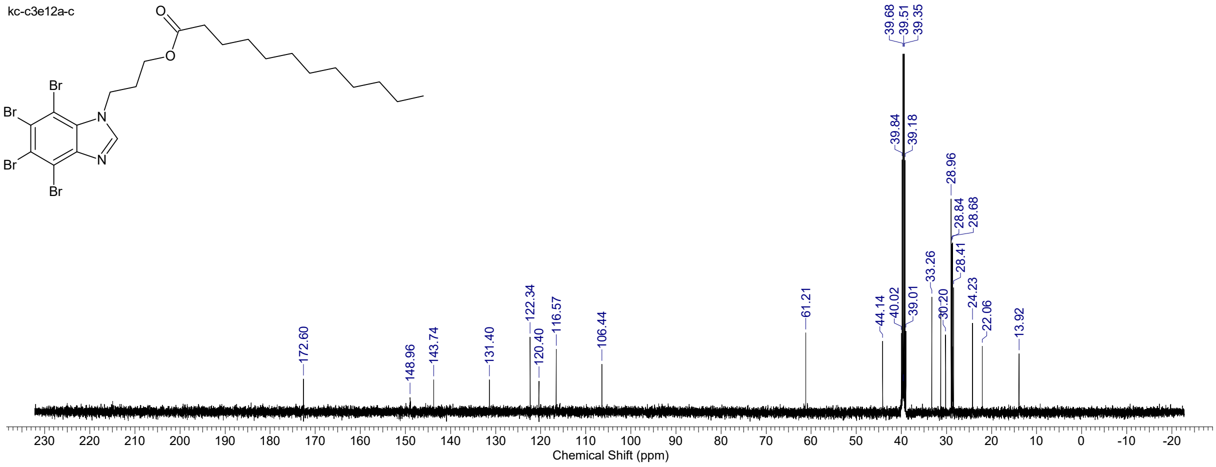

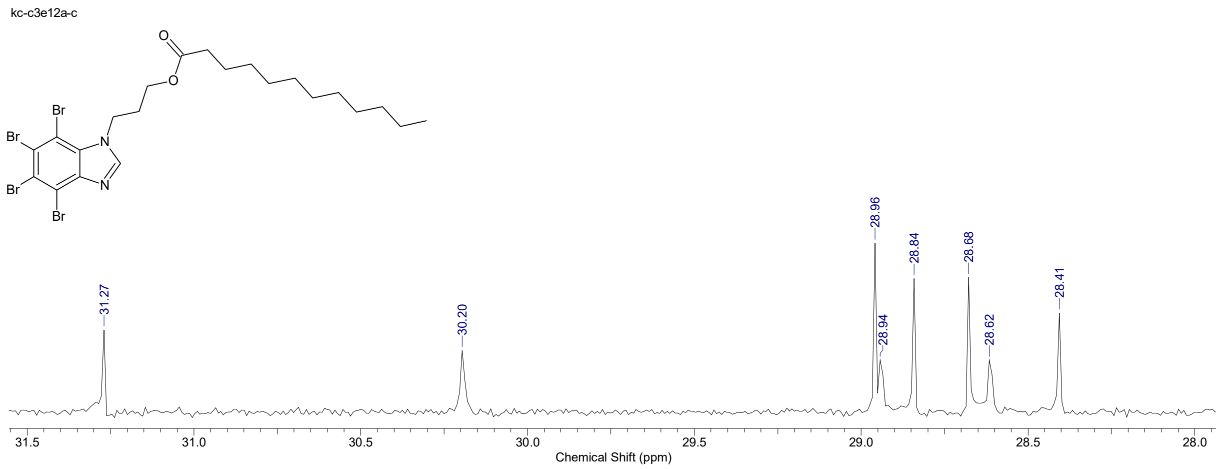

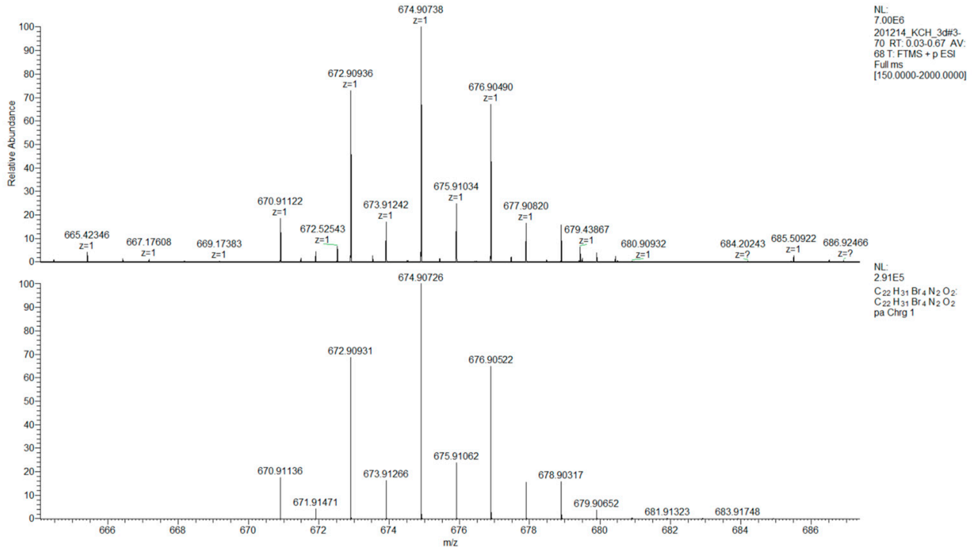

1.5 3-(4,5,6,7-Tetrabromo-2-methyl-1H-benzimidazol-1-yl)propan-1-ol (5)

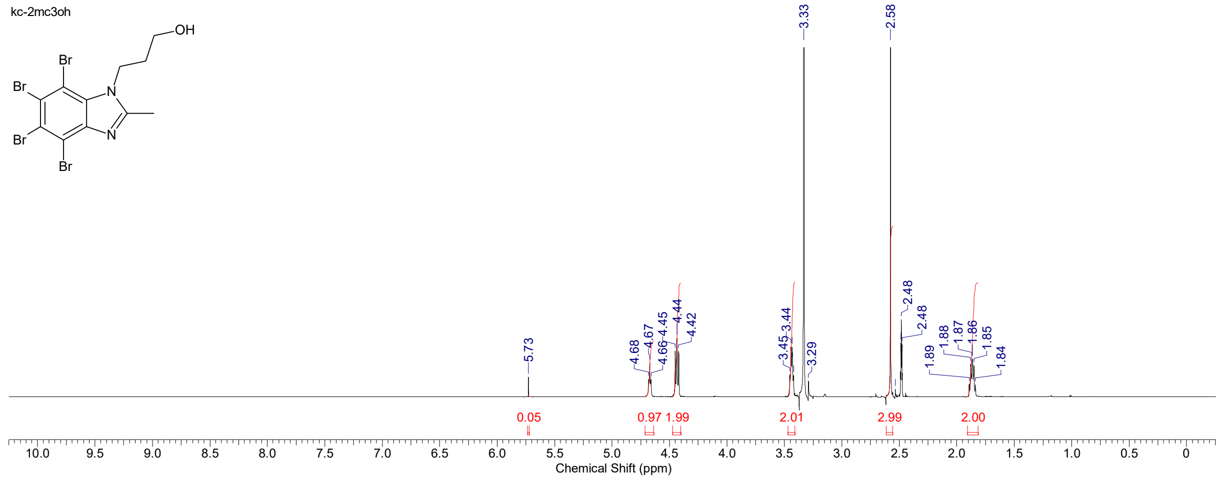

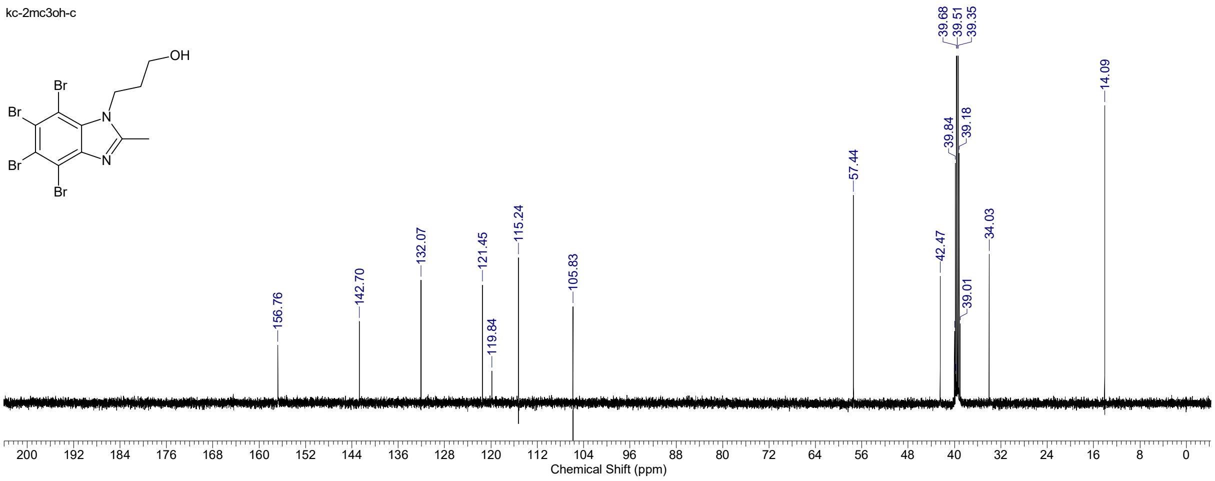

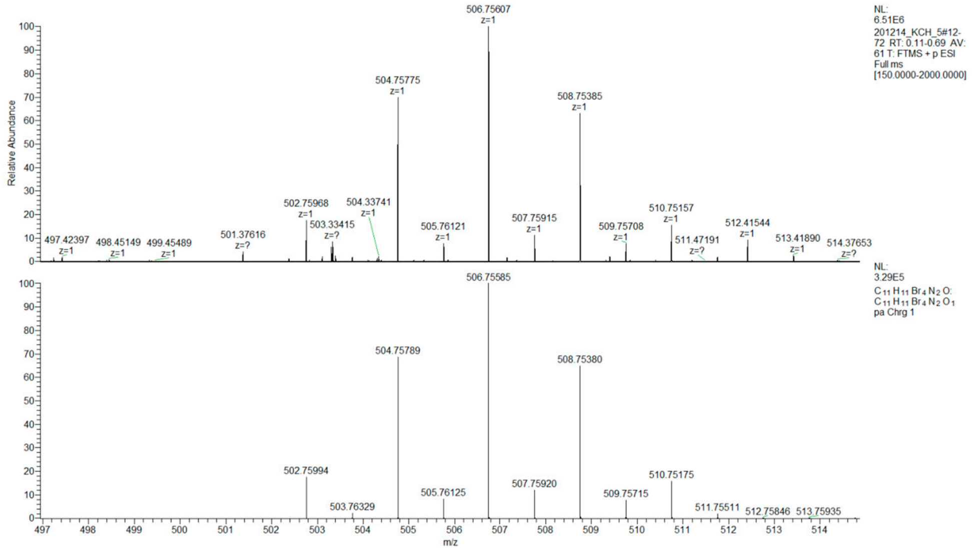

1.6 3-(4,5,6,7-Tetrabromo-2-methyl-1H-benzimidazol-1-yl)propyl butanoate (6a)

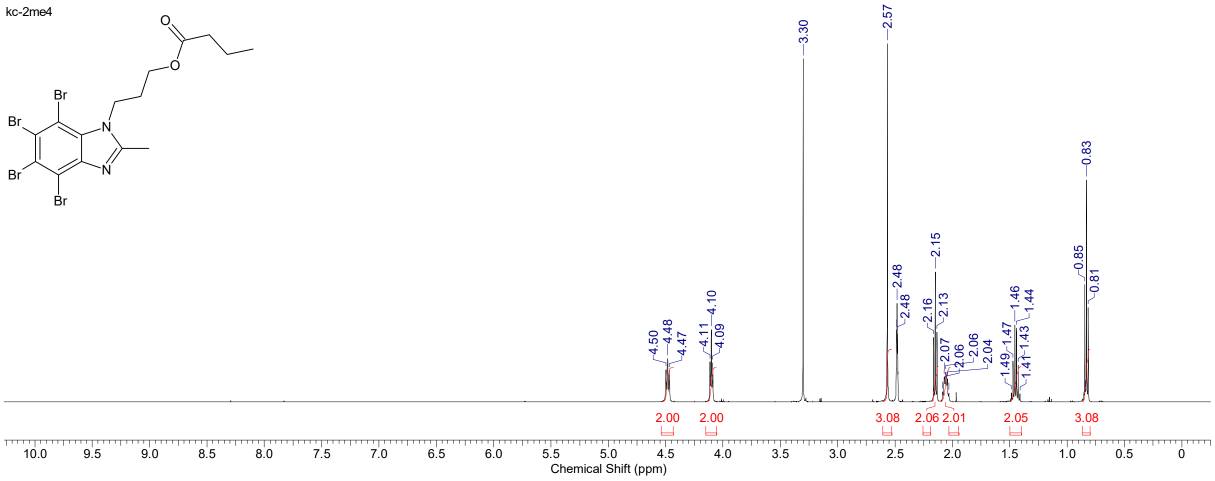

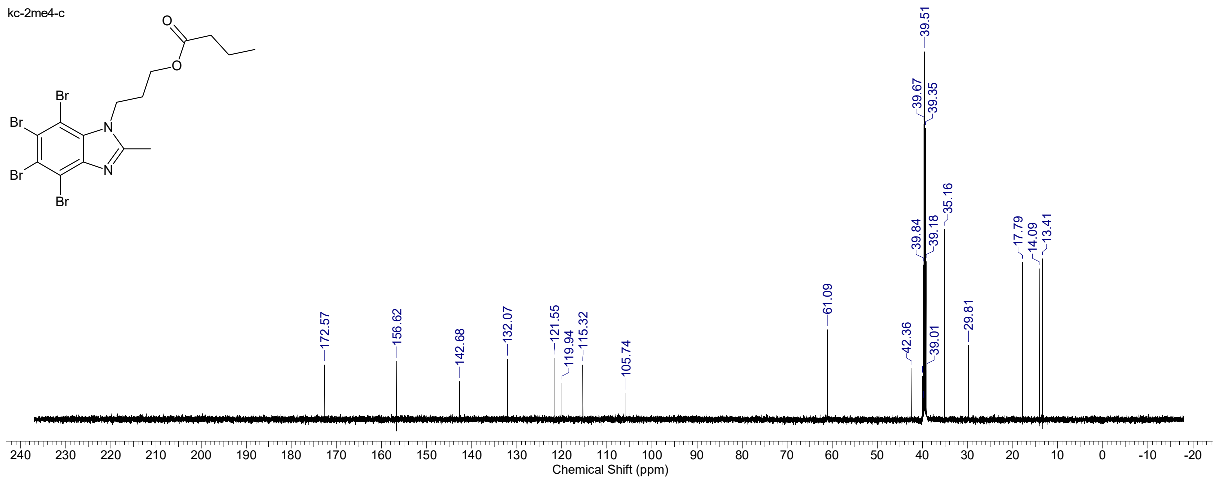

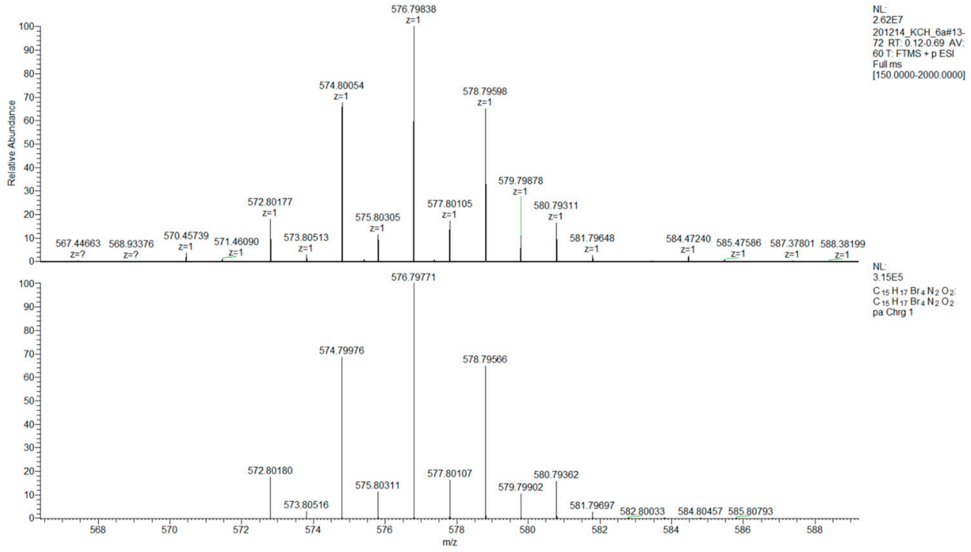

1.7 3-(4,5,6,7-Tetrabromo-2-methyl-1H-benzimidazol-1-yl)propyl hexanoate (6b)

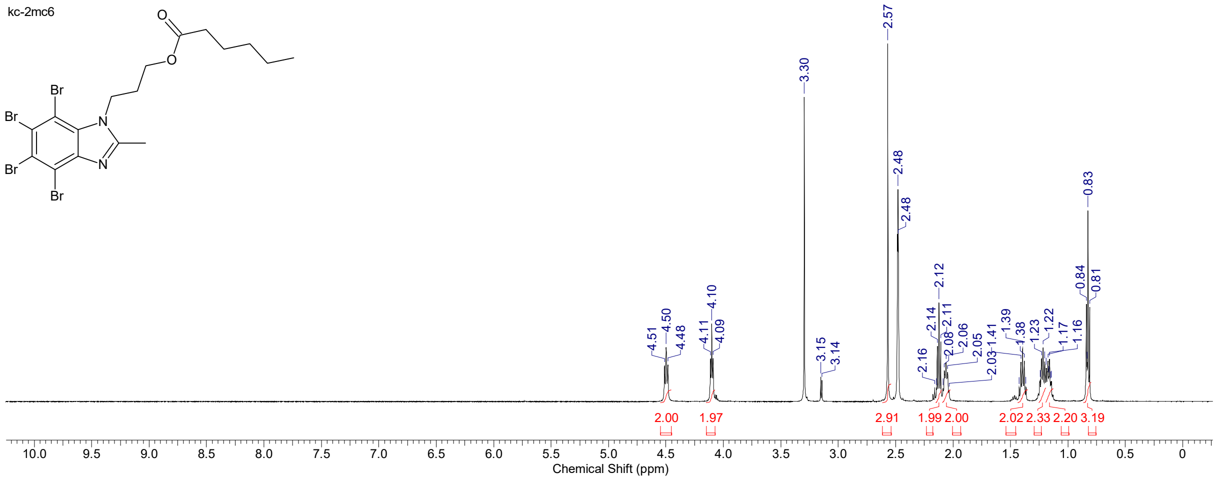

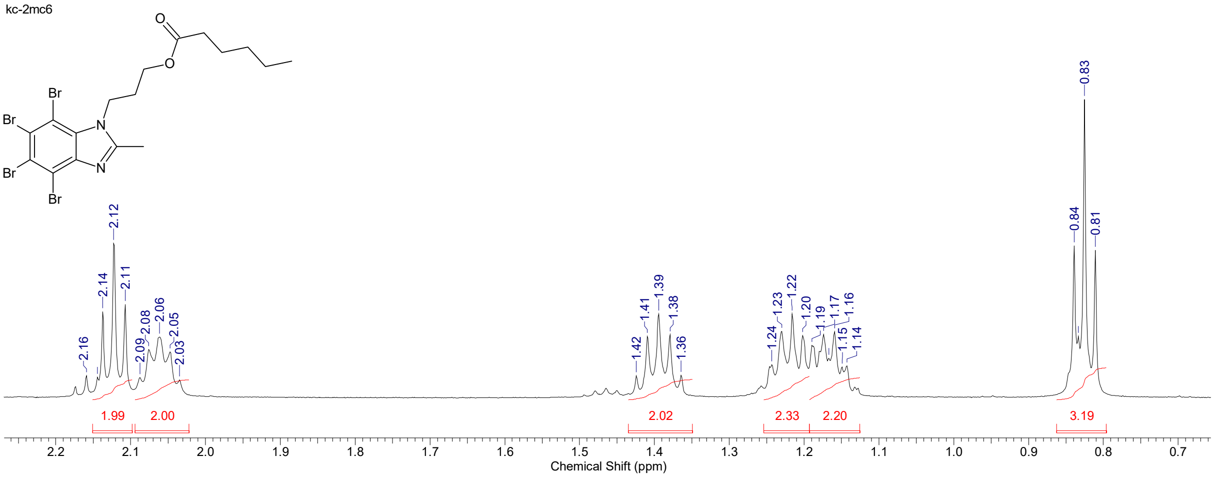
kc-2mc6
Br
N
O
2.16 2.14 2.12 2.11 2.09 2.08 2.06 2.05 2.03
1.42 1.41 1.39 1.38 1.36
1.24 1.23 1.22 1.20 1.19 1.17 1.16 1.15 1.14
0.84 0.83 0.81
1.99 2.00 2.02 2.33 2.20 3.19
Chemical Shift (ppm)

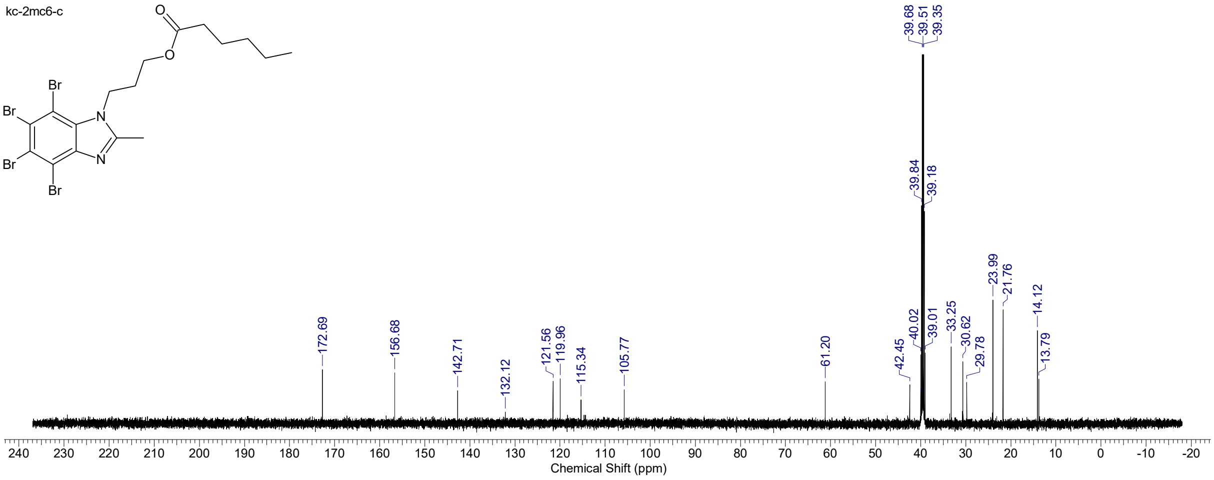
kc-2mc6-c
Br
N
O
172.69 156.68 142.71 132.12 121.56 119.96 115.34 105.77 61.20
42.45 40.02 39.84 39.68 39.51 39.35 39.18 39.01 33.25 30.62 29.78 23.99 21.76 14.12 13.79
Chemical Shift (ppm)

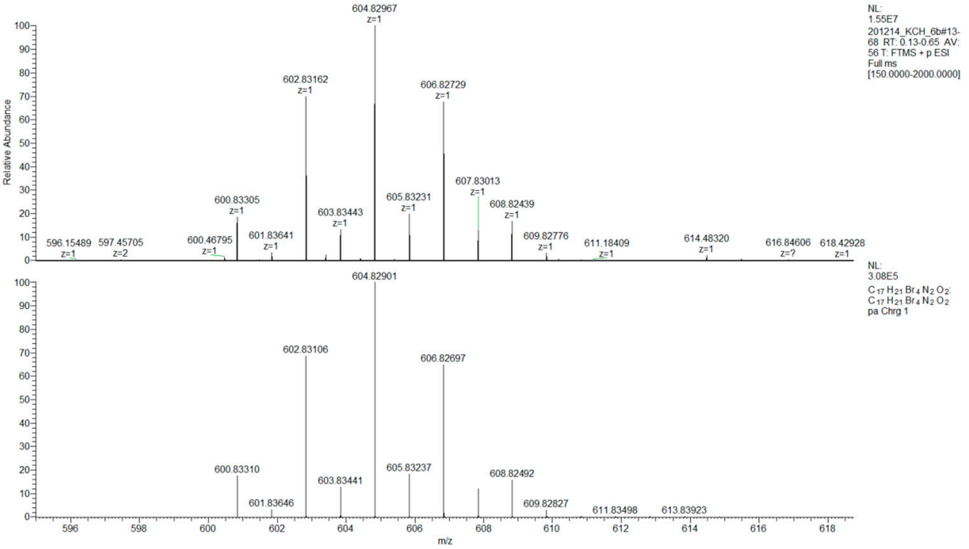
NL:
1.55E7
201214_KCH_6b#13-68 RT: 0.13-0.65 AV: 56 T: FTMS + p ESI Full ms [150.0000-2000.0000]
604.82967 z=1
602.83162 z=1
606.82729 z=1
607.83013 z=1
608.82439 z=1
600.83305 z=1
605.83231 z=1
603.83443 z=1
601.83641 z=1
609.82776 z=1
611.18409 z=1
614.48320 z=1
616.84606 z=?
618.42928 z=1
596.15489 z=1
597.45705 z=2
600.46795 z=1
Relative Abundance
NL:
3.08E5
C17 H21 Br4 N2 O2:
C17 H21 Br4 N2 O2
pa Chrg 1
604.82901
602.83106
606.82697
600.83310
605.83237
608.82492
603.83441
601.83646
609.82827
611.83498
613.83923
m/z

1.8 3-(4,5,6,7-Tetrabromo-2-methyl-1H-benzimidazol-1-yl)propyl octanoate (6c)

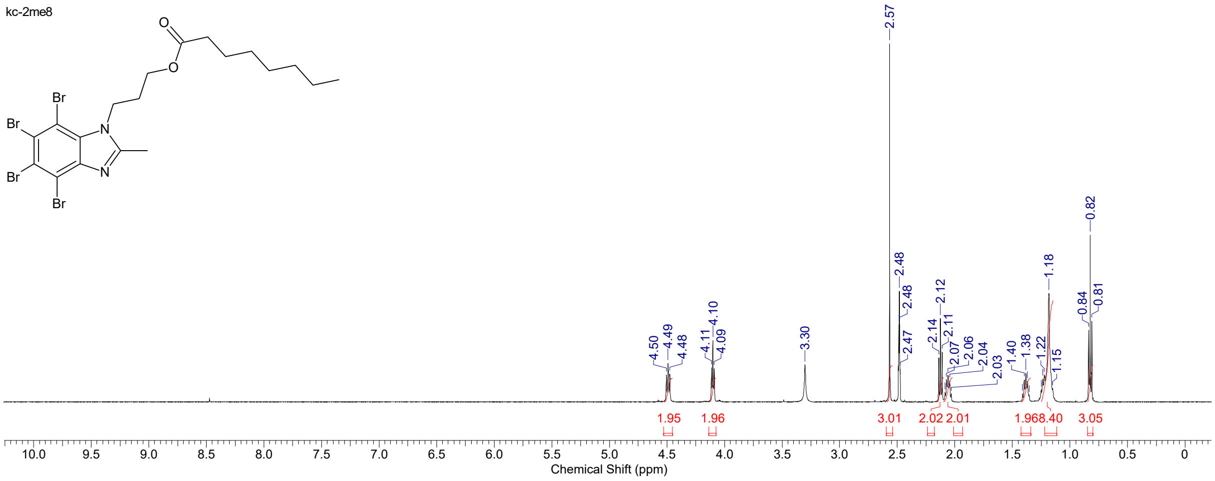

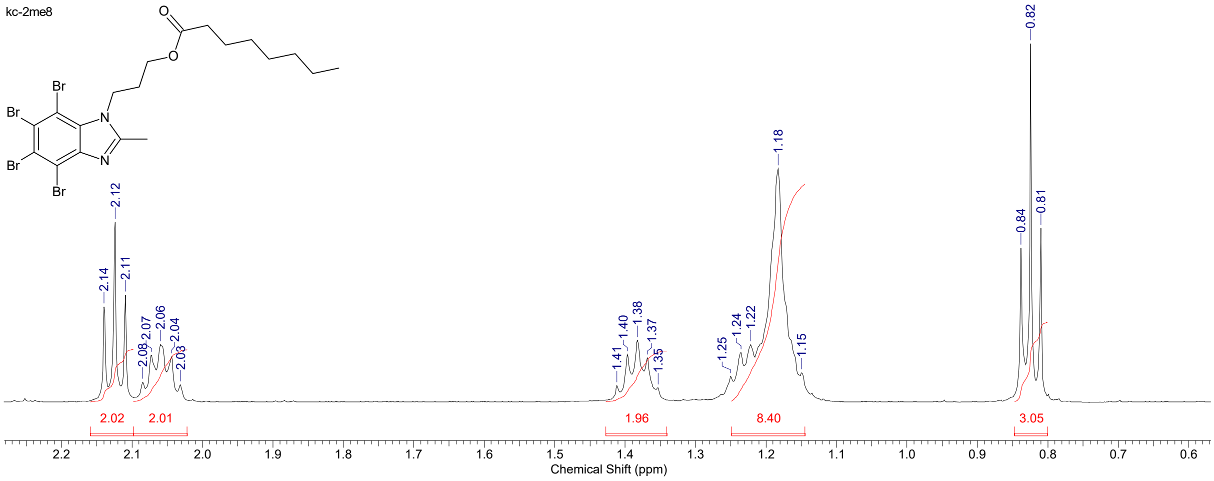

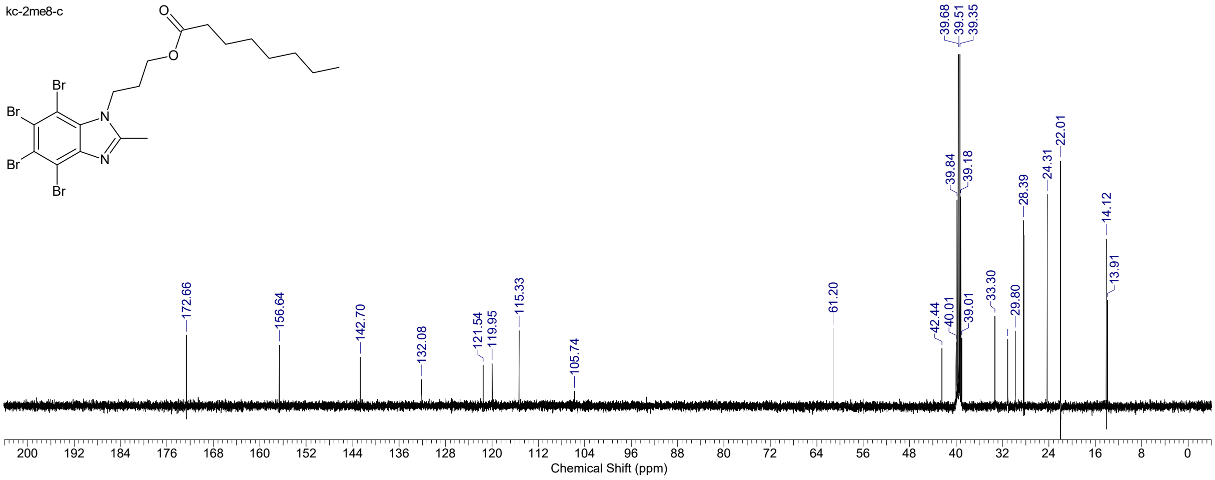

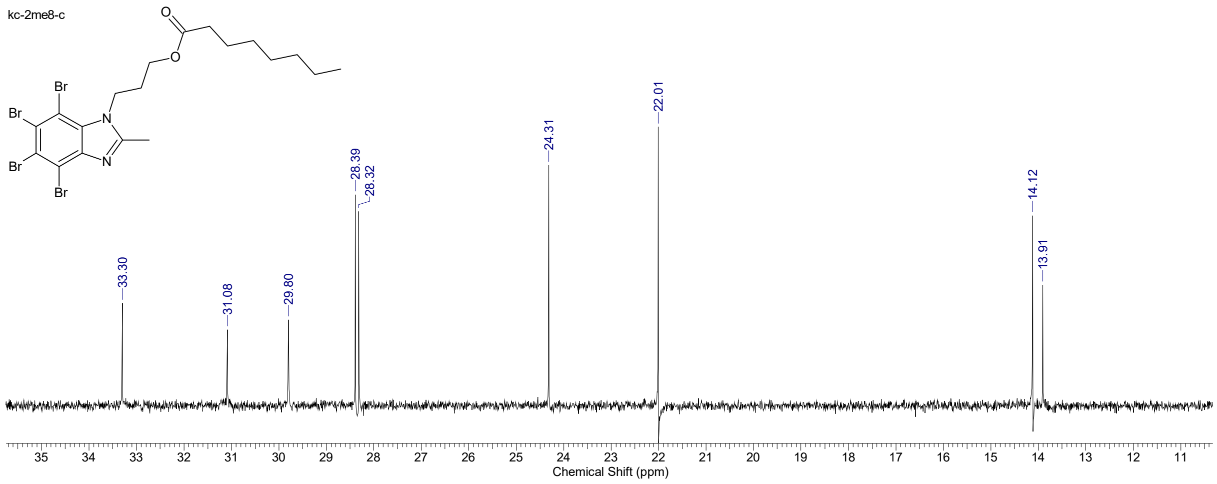

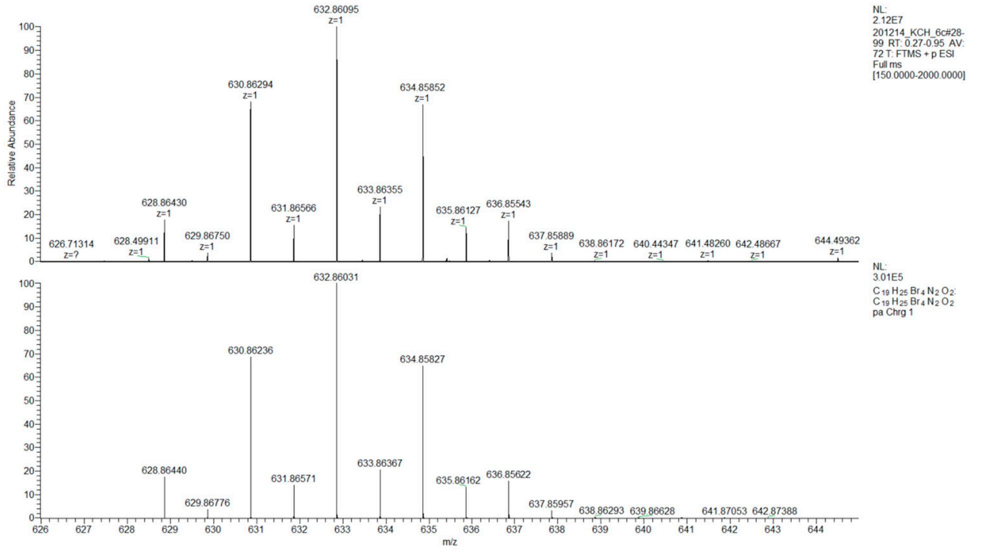

1.9 3-(4,5,6,7-Tetrabromo-2-methyl-1H-benzimidazol-1-yl)propyl dodecanoate (**6d**)

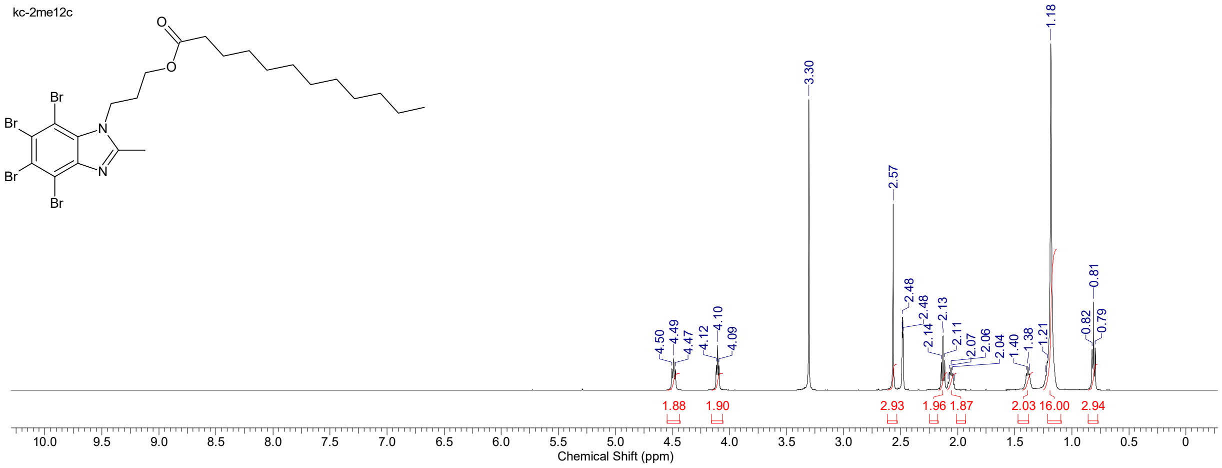

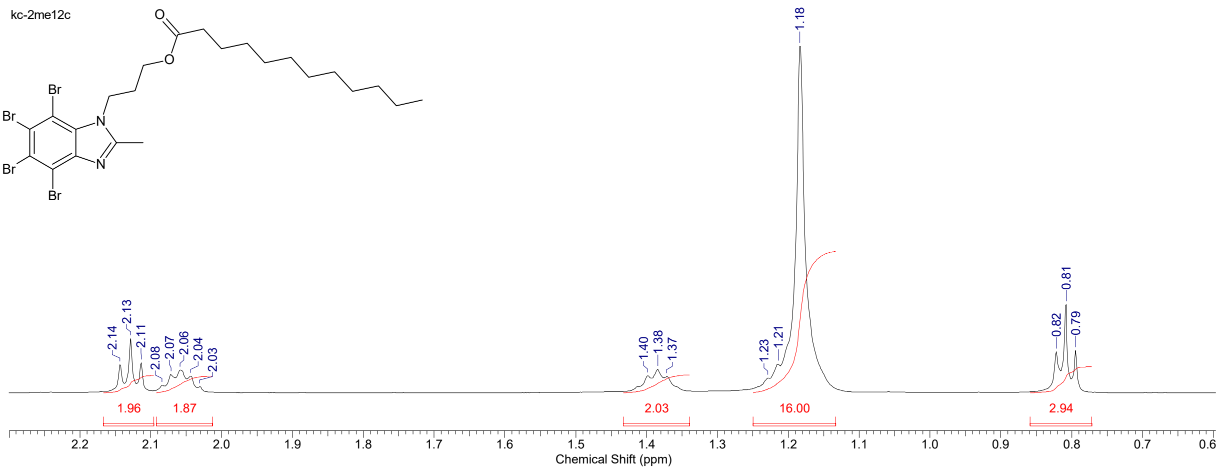
kc-2me12c
O
O
Br
Br
N
Br
N
Br
1.18
2.14
2.13
2.11
2.08
2.07
2.06
2.04
2.03
1.40
1.38
1.37
1.23
1.21
0.82
0.81
0.79
1.96
1.87
2.03
16.00
2.94
2.2 2.1 2.0 1.9 1.8 1.7 1.6 1.5 1.4 1.3 1.2 1.1 1.0 0.9 0.8 0.7 0.6
Chemical Shift (ppm)

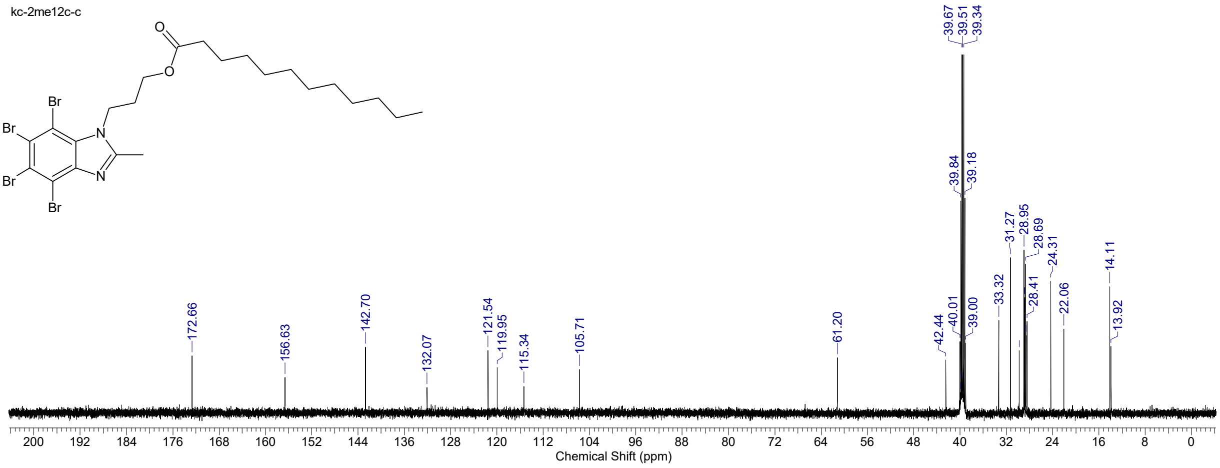
kc-2me12c-c
O
O
Br
Br
N
Br
N
Br
39.67
39.51
39.34
39.84
39.18
31.27
28.95
28.69
24.31
14.11
33.32
28.41
22.06
13.92
42.44
40.01
39.00
172.66
156.63
142.70
132.07
121.54
119.95
115.34
105.71
61.20
200 192 184 176 168 160 152 144 136 128 120 112 104 96 88 80 72 64 56 48 40 32 24 16 8 0
Chemical Shift (ppm)

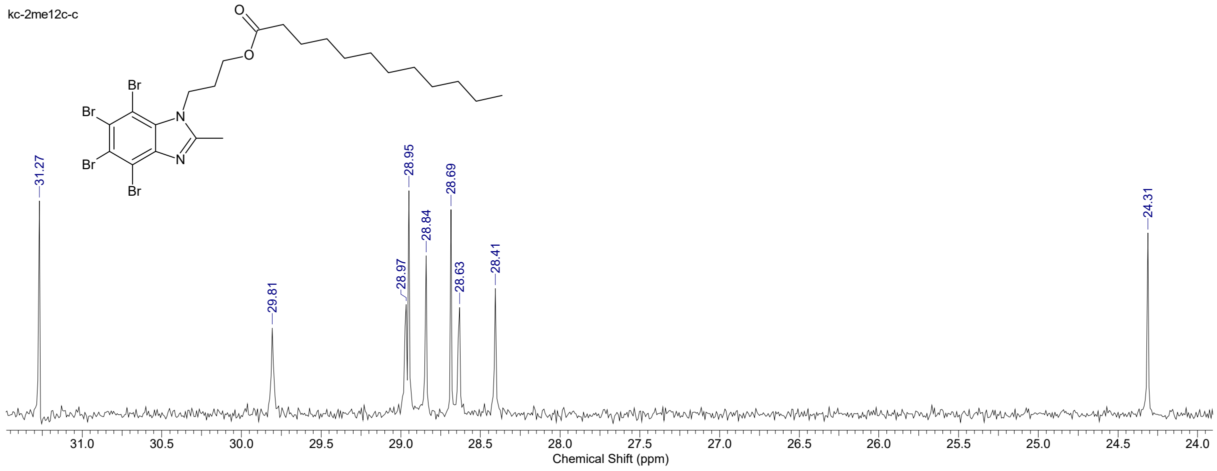
kc-2me12c-c
O
O
Br
Br
N
Br
N
Br
31.27
29.81
28.97
28.95
28.84
28.69
28.63
28.41
24.31
31.0 30.5 30.0 29.5 29.0 28.5 28.0 27.5 27.0 26.5 26.0 25.5 25.0 24.5 24.0
Chemical Shift (ppm)

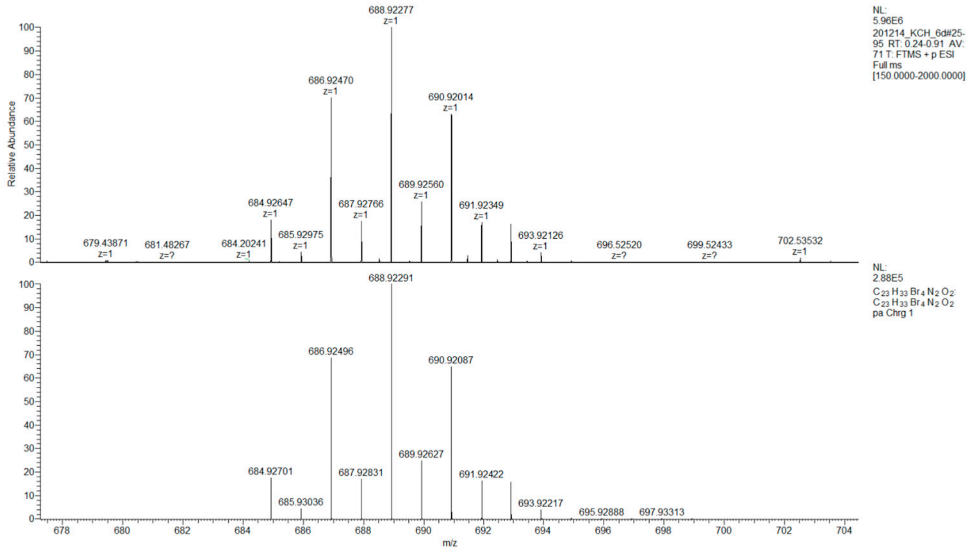

NL:
5.96E6
201214_KCH_6d#25-95 RT: 0.24-0.91 AV: 71 T: FTMS + p ESI Full ms [150.0000-2000.0000]
NL:
2.88E5
C23 H33 Br4 N2 O2:
C23 H33 Br4 N2 O2
pa Chrg 1
Relative Abundance
m/z
688.92277 z=1
686.92470 z=1
690.92014 z=1
689.92560 z=1
684.92647 z=1
687.92766 z=1
691.92349 z=1
685.92975 z=1
693.92126 z=1
679.43871 z=1
681.48267 z=?
684.20241 z=1
696.52520 z=?
699.52433 z=?
702.53532 z=1
688.92291
686.92496
690.92087
689.92627
684.92701
687.92831
691.92422
685.93036
693.92217
695.92888
697.93313

2. Inhibition of CK2 and PIM1 kinases by compounds 2, 4 and 5

2 CK2$\alpha_2\beta_2$

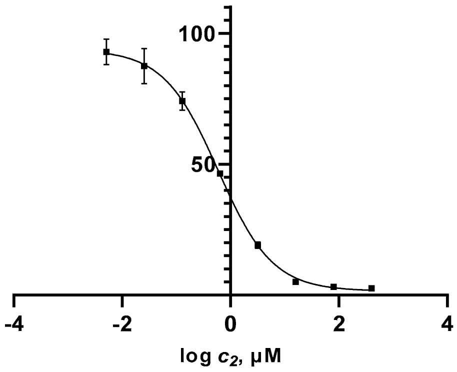

2 PIM1

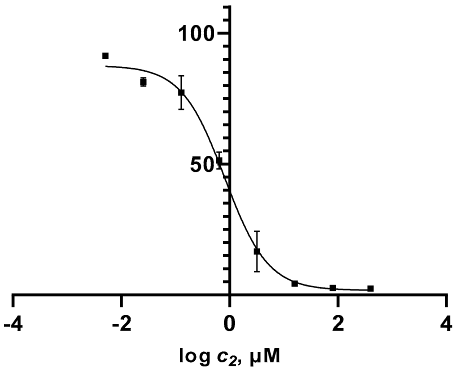

4 CK2α

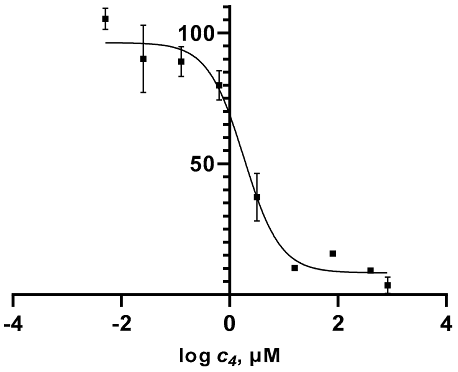

4 CK2$\alpha_2\beta_2$

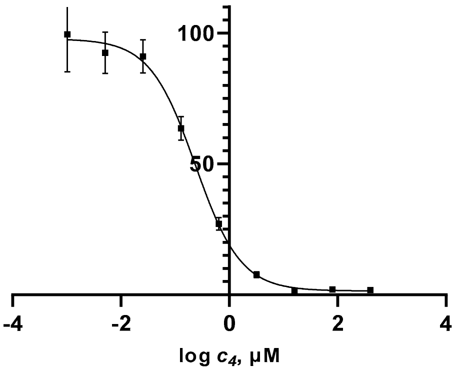

4 PIM1

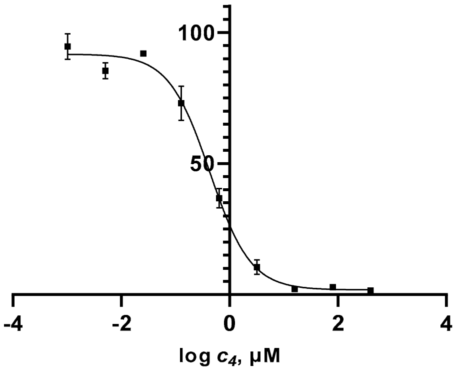

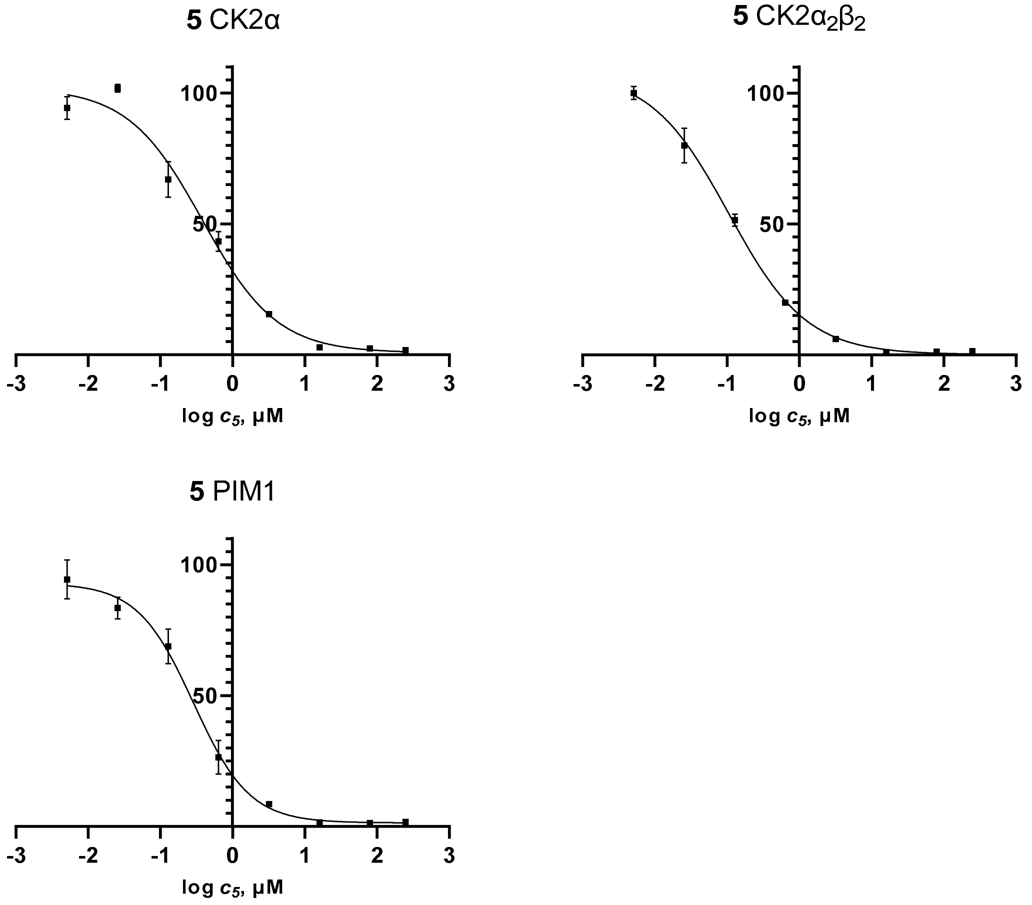

Fig. S1. Sigmoidal dose-response curves. Inhibition of human CK2 catalytic subunit (CK2α), CK2 holoenzyme (CK2 $\alpha_2\beta_2$) and PIM1 kinases by the previously and newly synthesized compounds was evaluated using radiometric assay. The synthetic peptide RRRADDSDDDDD was used as the substrate of CK2 and peptide ARKRRRHPSGPPTA as the substrate of PIM1. The experimental data were fitted to sigmoidal dose-response (variable slope) Y = Bottom + (Top-Bottom) / (1+10^((LogIC50-X)*HillSlope) equation in GraphPad Prism.

3. Docking studies

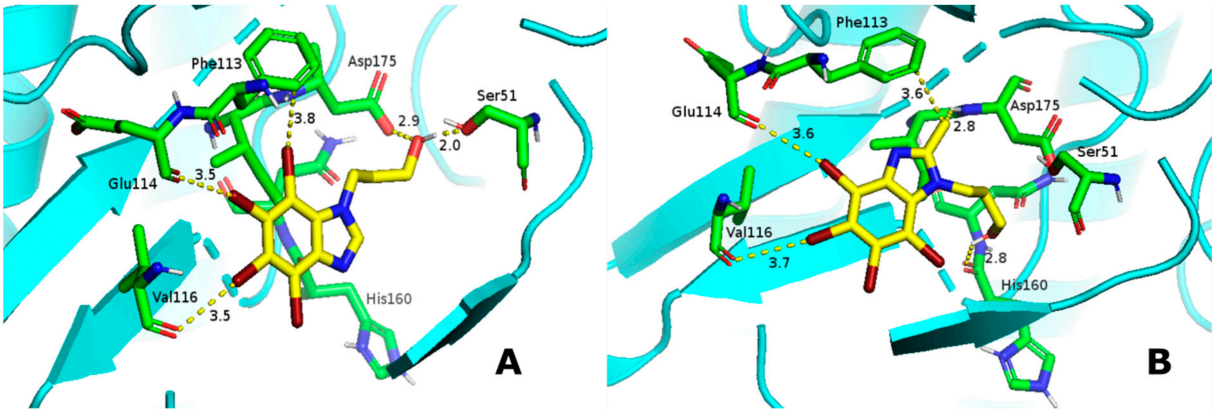

Fig. S2. Proposed binding models of **2** (**A**) and **5** (**B**) in the active site of CK2α (PDB: 7A4C, chain A). Molecular Docking was carried out using 1.1.2. AutoDock Vina program [1]. All ligands were drawn in s and saved as .mol2 files. The hydrogens and Gesteiger partial charges were added by 1.5.6. AutoDock tools [2] and the ligand files were saved in .pdbqt format. The crystal structure of human CK2α kinase was taken from Protein Data Bank with PDB code 7A4C chain A [3]. All water molecules and inhibitor (5,6,7-tribromo-1*H*-triazolo[4,5-*b*]pyridine) were removed, the polar hydrogen atoms were added and Gesteiger charges were calculated using AutoDock tools to get file in .pdbqt format. AutoGrid was used to find appropriate grid box size. The box centre was set at -5.000, -43.000 and 10.000 (x, y, z coordinates respectively) and final size space dimension x = 40 Å, y = 40 Å, z = 40 Å. Docking was performed with an exhaustiveness level of 32.

4. **Cytometry**

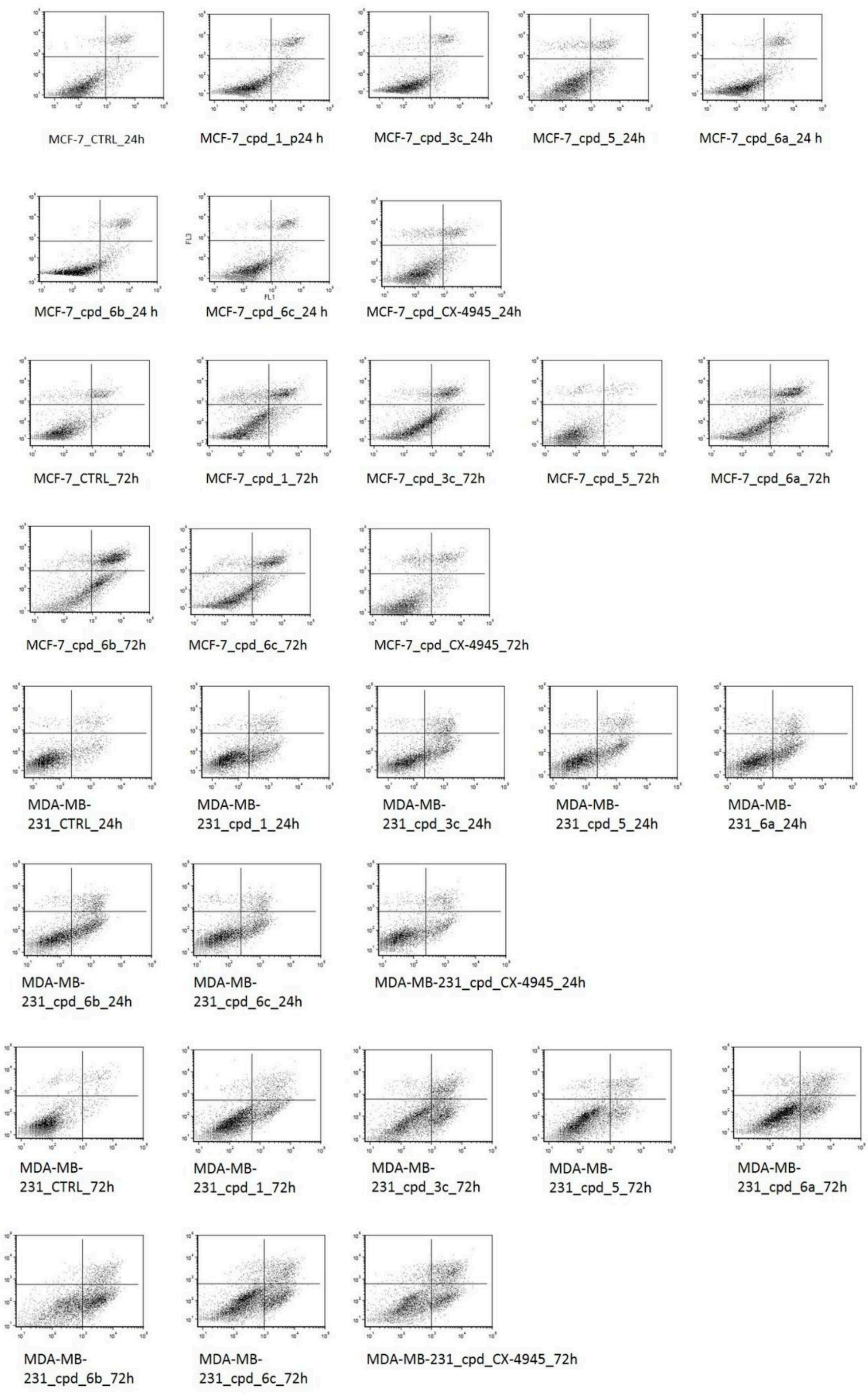

Fig. S3. Representative cytograms for MCF-7 and MDA-MB-231 cells treated with compounds **1**, **3c**, **5**, **6a-6c** for 24 h and 72 h of treatment. Cells were stained with annexin V-FITC and PI (propidium iodide).

The data were determined by CyFlow Cube 8, (Sysmex, Norderstedt, Germany) flow cytometer and analyzed with FCS Express 5 Flow software (De Novo Software, Glendale, CA, USA).

5. Contrast-phase microscopy

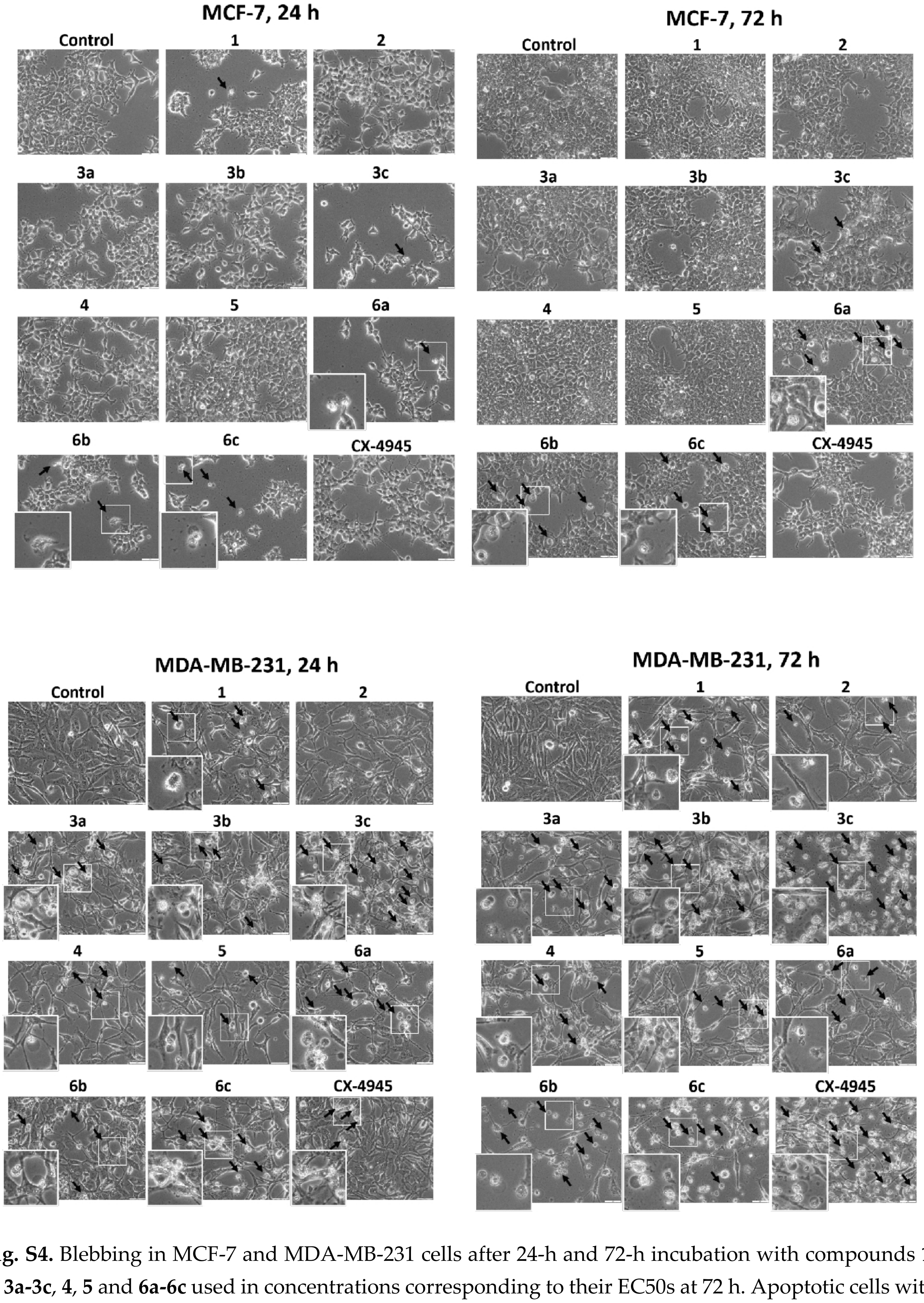

Fig. S4. Blebbing in MCF-7 and MDA-MB-231 cells after 24-h and 72-h incubation with compounds **1**, **2**, **3a-3c**, **4**, **5** and **6a-6c** used in concentrations corresponding to their EC50s at 72 h. Apoptotic cells with blebs were indicated with arrows. Phase-contrast images were made with the use of an inverted microscope Olympus CKX41 with Software: cellSens Dimension, and edited using Fiji distribution of the ImageJ 1.52a software.

6. Mycoplasma PCR ELISA

We used Mycoplasma PCR ELISA (Roche). According to manufacturer declaration the Kit detects following Mycoplasma, Acholeplasma and Ureaplasma species (*M. orale, M. arginini, M. fermentans, M. hyorhinis, M. salivarium, M. gallisepticum, M. hominis, M. bovis, M. californicum, M. bovoculi, M. Pg50 bovine group, M. bovigenitalium, M. hyopneumoniae, A. laidlawii, U. urealyticum*). DNA from other bacteria, yeast, and eukaryotic cells is not detectable. The procedure assumes amplification of a Mycoplasma-specific DNA sequence using PCR (with incorporated digoxigenin-labeled dUTP, followed by hybridization of the PCR-product to a Capture Probe immobilized one a plate and detection of the amplicon by ELISA using digoxigenin antibodies coupled to horseradish peroxidase (anti-DIG-POD), with the peroxidase-specific substrate TMB. Each test should be performed with one positive and two negative controls. Our results are presented in Table 1. Samples of culture supernatant were tested in duplicate.

Table S1. Mycoplasma PCR ELISA (Roche) results

Sample type	OD_{450}
Positive control	3,281
Negative K	0,065
Negative K	0,054
MDA	0,069
MDA	0,062
MCF	0,06
MCF	0,065

7. Detection of Cell Cycle Progression by Flow Cytometry

Table S2. The effect of compounds 1, 2, 3a–c, 4, 5 and 6a–c and CX-4945 on cell cycle progression in MCF-7 cells. MCF-7 cells were treated with the tested compounds used in concentrations corresponding to their EC50 for 72 h. Distribution of MCF-7 cells in different phases of the cell cycle were determined by flow cytometry and analyzed with FCS Express 5 Flow software to determine the percentage of cells in each phase of the cell cycle.

Cpd.	G1 (%)	S (%)	G2M (%)
CTRL	49.1	39.6	11.3
1	57.7	25.1	17.2
2	51.0	34.6	14.4
3a	57.8	28.4	13.8
3b	60.5	27.3	12.2
3c	59.4	29.6	11.0
4	53.6	29.7	16.7
5	60.2	26.3	13.5
6a	56.3	25.2	18.5
6b	50.1	30.0	19.9
6c	58.9	27.7	13.4
CX 4959	33.6	44.4	22.0

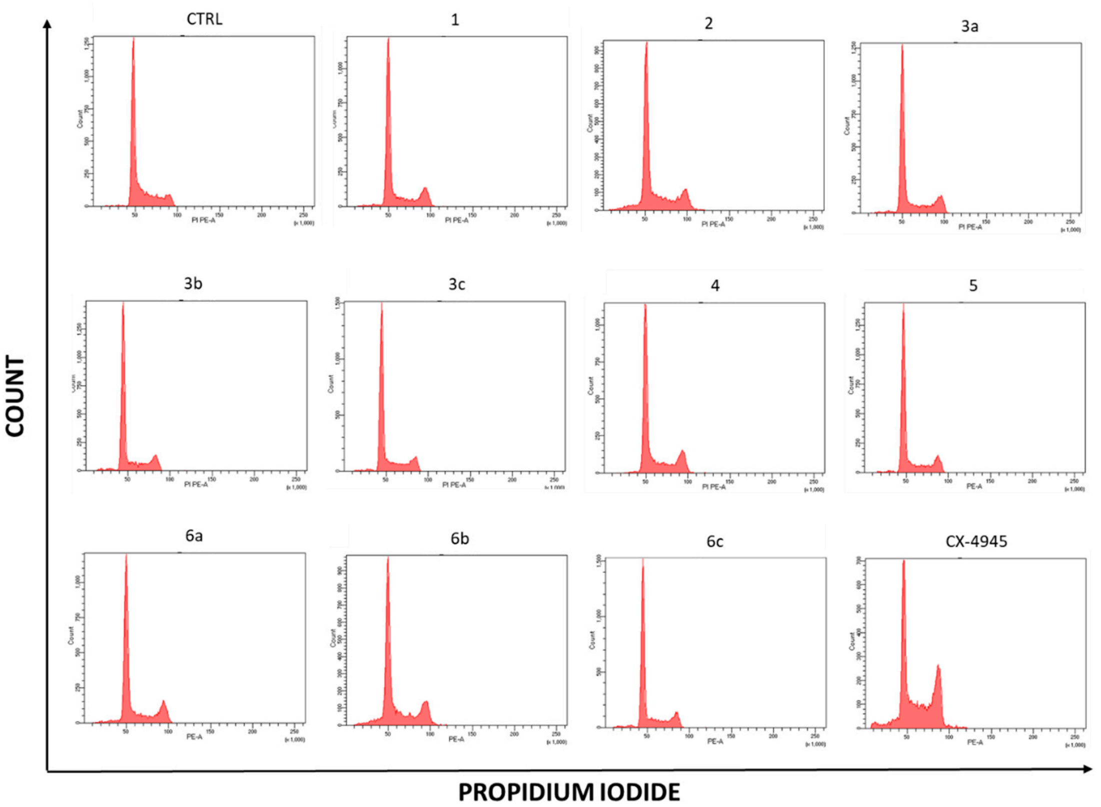

Fig. S5. DNA histograms of MCF-7 cell line treated with compounds 1, 2, 3a–c, 4, 5 and 6a–c and CX-4945 for 72 h. Distribution of MCF-7 cells in different phases of the cell cycle were determined by flow cytometry.

References:

[1] O. Trott, A. J. Olson, AutoDock Vina: Improving the Speed and Accuracy of Docking with a New Scoring Function, Efficient Optimization, and Multithreading. J. Comput. Chem. 31 (2010) 455-461. doi: 10.1002/jcc.21334.

[2] G. M. Morris, R. Huey, W. Lindstrom, M. F. Sanner, R. K. Belew, D. S. Goodsell, A. J. Olson, AutoDock4 and AutoDockTools4: Automated docking with selective receptor flexibility. J. Comput. Chem. 16 (2009) 2785-2791. doi: 10.1002/jcc.21256.

[3] K. Chojnacki, D. Lindenblatt, P. Winska, M. Wielechowska, C. Toelzer, K. Niefind, M. Bretner, Synthesis, biological properties and structural study of new halogenated azolo[4,5-b]pyridines as inhibitors of CK2 kinase. Bioorg. Chem. 106 (2021) 104502. doi: 10.1016/j.bioorg.2020.104502.
